# How to measure blood pressure using an arterial catheter: a systematic 5-step approach

**DOI:** 10.1186/s13054-020-02859-w

**Published:** 2020-04-24

**Authors:** Bernd Saugel, Karim Kouz, Agnes S. Meidert, Leonie Schulte-Uentrop, Stefano Romagnoli

**Affiliations:** 1 Department of Anesthesiology, Center of Anesthesiology and Intensive Care Medicine, University Medical Center Hamburg-Eppendorf, Martinistrasse 52, 20246 Hamburg, Germany; 2 Outcomes Research Consortium, Cleveland, OH USA; 3 Department of Anaesthesiology, University Hospital LMU Munich, Munich, Germany; 4 Department of Health Science, Section of Anesthesia and Critical Care, University of Florence, Florence, Italy; 5 Department of Anesthesia and Critical Care, Azienda Ospedaliero-Universitaria Careggi, Florence, Italy

**Keywords:** Cardiovascular dynamics, Hemodynamic monitoring, Patient monitoring, Critical care, Intensive care medicine, Anesthesia, Arterial line, Arterial pressure

## Abstract

Arterial blood pressure (BP) is a fundamental cardiovascular variable, is routinely measured in perioperative and intensive care medicine, and has a significant impact on patient management. The clinical reference method for BP monitoring in high-risk surgical patients and critically ill patients is continuous invasive BP measurement using an arterial catheter. A key prerequisite for correct invasive BP monitoring using an arterial catheter is an in-depth understanding of the measurement principle, of BP waveform quality criteria, and of common pitfalls that can falsify BP readings. Here, we describe how to place an arterial catheter, correctly measure BP, and identify and solve common pitfalls. We focus on 5 important steps, namely (1) how to choose the catheter insertion site, (2) how to choose the type of arterial catheter, (3) how to place the arterial catheter, (4) how to level and zero the transducer, and (5) how to check the quality of the BP waveform.

## Background

Arterial blood pressure (BP) is a fundamental cardiovascular variable. BP monitoring is mandatory in patients having surgery with anesthesia [1] and patients with circulatory shock [2]. In perioperative and intensive care medicine, BP measurements have a significant impact on patient management, especially for the timely and correct identification and treatment of hypotension [3, 4].

There are several methods to measure BP: intermittent non-invasive measurements using oscillometry, continuous non-invasive measurements using methods such as the vascular unloading technique (also called “finger-cuff technology”), or continuous invasive measurements using an arterial catheter (also referred to as “direct” BP measurement) [5, 6]. The choice of the BP monitoring method is important as it directly impacts clinical decision-making. Intermittent non-invasive measurements using oscillometry show clinically significant discrepancies compared to continuous invasive measurements using an arterial catheter and especially overestimate low BP [7, 8]. In addition, continuous invasive BP monitoring detects twice as much intraoperative hypotension than intermittent non-invasive measurements using oscillometry and triggers vasopressor therapy in adults having non-cardiac surgery [9]. Continuous non-invasive BP monitoring—compared to intermittent non-invasive measurements using oscillometry—also reduces the amount of intraoperative hypotension [10, 11]. However, validation studies comparing continuous non-invasive BP measurements to continuous invasive BP measurements using an arterial catheter revealed contradictory results regarding the interchangeability of the methods [12, 13]. In addition, although continuous non-invasive BP monitoring using finger-cuff technologies is a promising approach in patients having surgery, it is not recommended in critically ill patients with circulatory shock [14]. Therefore, invasive BP monitoring remains to be the clinical reference method to measure BP in perioperative and intensive care medicine.

Indications for the insertion of an arterial catheter include the need for continuous BP monitoring, the impracticality of non-invasive BP measurements, or the need for repeated arterial blood sampling. Additionally, advanced invasive hemodynamic monitoring (pulse wave analysis, transpulmonary thermodilution) requires an arterial catheter.

A major—but underestimated—risk of invasive BP monitoring using an arterial catheter is that wrong therapeutic actions are taken based on erroneous BP readings caused by unrecognized artifacts or measurement problems [15]. BP and advanced hemodynamic variables can only be reliably measured when BP waveforms are correctly recorded, transmitted, and analyzed. A key prerequisite for correct invasive BP monitoring using an arterial catheter is an in-depth understanding of the measurement principle, of BP waveform quality criteria, and of common pitfalls that can falsify BP readings. Therefore, we systematically describe how to place an arterial catheter, correctly measure BP, and identify and solve common pitfalls.

## How to measure blood pressure using an arterial catheter: a systematic 5-step approach

To correctly measure BP using an arterial catheter, we propose a systematic 5-step approach that helps to (1) choose the catheter insertion site, (2) choose the type of arterial catheter, (3) place the arterial catheter, (4) level and zero the transducer, and (5) check the quality of the BP waveform.

### Step 1: Catheter insertion sites

Commonly used anatomical sites for arterial catheter placement are the radial, brachial, and femoral arteries. Less frequently used insertion sites are the ulnar, axillary, temporal, posterior tibial, and dorsal pedis arteries [16]. Contraindications for arterial cannulation are local infection, thrombosis, active Raynaud’s syndrome, thromboangiitis obliterans, or abnormalities in vessel anatomy at the puncture site.

Catheter insertion in the radial artery is most commonly used because it is technically easy and rarely associated with major complications [17]. The radial artery is best palpated between the distal radius and the tendon of the flexor carpi radialis 1–2 cm proximal from the wrist. Performing modified Allen’s test to test the collateral circulation has poor diagnostic accuracy and is not considered a reliable predictor of ischemic complications following cannulation of the radial artery [18]. Using technical methods such as pulse oximetry, plethysmography, or Doppler ultrasound (US) when performing modified Allen’s test does not substantially improve its accuracy [19].

For radial artery cannulation, the wrist and hand should be carefully immobilized and secured with the wrist resting across a soft support and mildly dorsiflexed to keep the artery in position. Cannulation should be started as distally as possible, as one can move to a more proximal puncture site after unsuccessful cannulation. The ulnar artery may be safely cannulated even following failed attempts to access the ipsilateral radial artery [20, 21].

The brachial artery, even though it is the main artery of the arm, can also be used for BP monitoring [22]. Although the placement of an arterial catheter into the brachial artery has a low overall complication rate of 0.2%, its complications are associated with long hospitalization and even increased mortality [23]. The brachial artery is best palpated medial to the biceps tendon in the antecubital fossa, when the shoulder is slightly abducted, the elbow extended, and the forearm supinated.

The femoral artery is the largest artery used for arterial catheter placement, and the complication rate of arterial catheter placement in the femoral artery is comparable to those of other sites [17]. The femoral artery is best palpated just below the midpoint of the inguinal ligament with the patient lying supine and the patient’s leg being extended, slightly abducted, and externally rotated. Puncture of the femoral artery should be performed distally to the inguinal ligament to minimize the risk of hemorrhage into the pelvis or retroperitoneum.

The axillary artery is the only alternative to the femoral artery for central BP measurement. In some situations, the axillary artery may be preferred over the femoral artery (e.g., obesity, ilio-femoral vascular disease, and lower extremity edema) [24]. The axillary artery can be palpated best when the arm is abducted and externally rotated. The cannulation site should be as high into the apex of the axilla as possible.

The morphology of the BP waveform changes when the BP wave moves from the aorta to a more peripheral artery due to pulse wave amplification phenomena. In the periphery, the BP waveform shows a higher systolic BP, a steeper slope of the systolic upstroke, a lower diastolic BP, and a lower and later dicrotic notch compared to BP waveforms recoded at the aortic root [25]. The decrease in diastolic BP is less pronounced than the increase in systolic BP [25].

### Step 2: Choosing the type of arterial catheter

The choice of the type of the arterial catheter depends on several factors, including the artery to be cannulated and expected cannulation problems.

The catheter length should be chosen primarily depending on the cannulation site. When using the brachial, femoral, or axillary artery, a longer catheter is recommended because of the distance between the surface of the skin and the lumen of the artery; using longer arterial catheters reduces the risk of dislocation. The length and inner diameter of the catheter influence the damping properties of the BP measurement system (for details regarding the dynamic response of the pressure transducer, see step 5). Twenty gauge catheters have been shown to be less affected by underdamping than 18-G catheters [15, 26] and can be generally recommended for radial cannulation. Complications occur less often with 20-G catheters compared to larger catheters [16].

In difficult situations (e.g., edema, vascular sclerosis, obesity, cannulation during cardiopulmonary resuscitation) or after unsuccessful direct puncture, a separate or integral guidewire approach is recommended, as it is the technique with the highest success rates [27, 28].

### Step 3: Placement of the arterial catheter

Before cannulation, the equipment for arterial catheter placement needs to be carefully prepared. It includes sterile gloves and drapes, surgical mask, alcohol-based antiseptic skin prep solutions based on chlorhexidine gluconate [29, 30], arterial catheter, adhesive tape, tubing system, and transducer kit. The tubing system has to be filled with crystalloid fluid and connected to a soft bag placed into a pressure bag set at 300 mmHg. This pressure prevents the backflow of blood from the cannulated artery into the catheter and the transducer system and maintains a continuous column of fluid from the system into the artery. Heparinized solutions are not recommended because heparin exposure might promote antibody formation leading to heparin-induced thrombocytopenia [31].

The insertion of the arterial catheter has to be performed under sterile conditions. Therefore, the skin is prepared with an antiseptic solution (and local anesthetic should be subcutaneously administered above the artery in conscious patients).

Different techniques can be used to place the catheter with or without the use of US [32], namely the separate guidewire approach, integral guidewire approach, and direct puncture.

#### Separate guidewire approach

- “Seldinger” technique

The needle is advanced at a 30 to 45° angle to the skin towards the point where the pulse is palpated through the vessel. As soon as the artery is punctured, indicated by pulsatile blood flow through the needle, the guidewire is introduced through the lumen of the needle. After the needle is removed, the catheter is advanced over the guidewire. The guidewire is then removed, leaving only the catheter in place.

b) “Over-the-wire” technique (“modified Seldinger” technique)

The arterial catheter with an inner needle is inserted at a 30 to 45° angle to the skin. As soon as the artery is punctured, the blood fills the hub of the catheter. The needle-catheter is then advanced slightly through the vessel, the needle is completely removed, and the catheter is slowly withdrawn until pulsatile blood flow is observed. Then, the separate guidewire is advanced into the vessel through the catheter. As a next step, the catheter is advanced over the wire, and the guidewire is removed, leaving only the catheter in place.

#### Integral guidewire approach (“modified Seldinger” technique)

This approach uses an integral guidewire that is inseparable from the catheter kit. After inserting the needle-guidewire-catheter unit at a 30 to 45° angle to the skin, it is advanced slowly until pulsatile blood flow is observed. Then, the angle of the needle-guidewire-catheter unit is decreased, bringing it more parallel to the skin. The guidewire tab is advanced into the artery through the needle and catheter. Then, the catheter is advanced into the artery over the needle and guidewire, and the needle-guidewire component of the unit is removed.

#### Direct puncture (“over-the-needle” technique)

The needle-catheter unit is advanced at a 30 to 45° angle to the skin. When the blood flows towards the hub after puncture of the artery, the needle-catheter must be slightly advanced and lowered to a 10 to 15° angle to the skin. This has to be done, as the needle is slightly longer than the catheter and the backflow of blood just indicates that the needle tip—and not implicitly the catheter—is in the vessel. Then, the catheter is advanced into the artery and the needle is removed.

After placement of the catheter, it is connected to the transducer system and secured with suture or in a sutureless fashion with an adhesive dressing.

#### Ultrasound-guided approach

All of the arterial cannulation techniques described above can be performed under US guidance. Although several medical societies distinctly recommend the use of US for central venous catheterization [33, 34], current guidelines do not (yet) recommend routine use of US for arterial catheterization [35, 36]. Two recently published meta-analyses of randomized controlled trials comparing radial arterial cannulation using the landmark technique with US-guided techniques in adults provided evidence that US techniques offer advantages with regard to first-pass success and failure rate [37, 38]. It seems obvious that—after education and training—arterial catheter placement under real-time visualization has advantages over the landmark technique. In specific situations, the use of US can facilitate successful arterial access (e.g., challenging puncture, limited access sites, difficulties in palpating the pulse, or after failed puncture attempts) [35, 39]. US-guided arterial catheter placement has to be performed under sterile conditions with a sterile cover for the US probe and a sterile conductive medium [40].

Different US-guided arterial catheter placement techniques have been described [41]. The static or indirect technique is applied to identify the target artery before puncture (i.e., “US-assisted”), while the real-time or direct arterial catheter placement technique is performed under continuous US control (i.e., “US-guided”).

Short- and long-axis views (depending on the orientation of the US probe relative to the vessel) can be used for arterial catheter placement. For the short-axis (out-of-plane) technique, the US probe is placed orthogonal to the artery, so that the cross-sectional area of the arterial lumen is visualized. Thereby, it is not possible to differentiate between the needle tip and the needle shaft, because the needle/catheter is visualized as a point on the US screen. To circumvent this disadvantage, a modified short-axis technique (dynamic needle tip positioning) can be performed, in which the needle is gradually advanced with stepwise adjustment of the US probe following the needle tip until it is visible in the vessel lumen [42]. When using the long-axis (in-plane) technique, the US probe is oriented parallel to the artery, and the needle/catheter can be completely visualized. This technique may be more difficult to learn, but once it is mastered, it is superior to the short-axis approaches [39].

#### Complications of arterial catheter placement

Common complications of arterial catheter placement include local pain and paresthesia, hematoma, and minor bleeding. The risk of ischemic complications is less than 0.1% [17]. Major, but less common, complications of arterial catheter placement are major bleeding, embolism of air or thrombotic material, vascular thrombosis and occlusion, vessel injury, pseudoaneurysm formation, and local nerve injury. Temporary occlusion of the femoral artery occurs in 1% of cases [17]. It is less frequently compared to the incidence of radial artery occlusion (1.5% up to 35%) [17]. Permanent occlusion of the radial artery however appears to be rare (mean incidence, 0.09%) [17]; because of collateral recruitment, this usually does not lead to ischemic complications [20]. Pseudoaneurysm formation of the femoral artery due to arterial cannulation occurs similarly often (0.3%) compared to the radial (0.09%) and axillary artery (0.1%) [17]. The use of an arterial catheter bears the risk of unintentional intra-arterial injection of medications, disconnection of the tubing system resulting in massive blood loss, and catheter-related bloodstream infections [43]. The rates of catheter-related bloodstream infections are higher for femoral artery cannulation compared to radial artery cannulation (relative risk, 1.93) [43].

### Step 4: Leveling and zeroing of the pressure transducer

The pressure transducer (where the mechanical signal is transduced into an electrical signal [44]) must be leveled and zeroed to ensure that BP measurements are accurate. It needs to be distinguished between a measurement using a transducer alone without a zero line or a transducer with a zero line, as the leveling and zeroing procedures differ between the two methods.

**Fig. 1:**
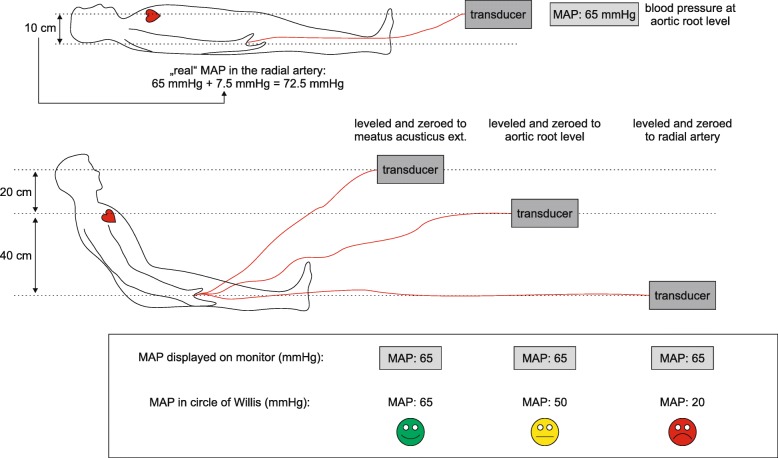
Leveling and zeroing of the pressure transducer. A vertical difference of 10 cm between the pressure transducer and the artery of interest results in a pressure difference of 7.5 mmHg due to hydrostatic pressure. Especially in non-supine positions (e.g., beach chair), wrong leveling and zeroing can result in wrong therapeutic actions with the consequence of low perfusion pressure in the circle of Willis

When using a transducer without a zero line, the transducer—or more precisely the stopcock of the transducer opening towards atmospheric pressure—needs to be leveled to the level of the vessel of interest (Fig. 1). The correct leveling of the transducer is of crucial importance, as a height difference between the transducer level and the level of the vessel of interest of only 10 cm results in a pressure difference of 7.5 mmHg because of the hydrostatic pressure difference. For instance, if a patient has surgery in a beach-chair position (i.e., sitting position—for example, during shoulder surgery) and if the clinician wants to monitor BP at the aortic root level, the transducer must be leveled to the phlebostatic axis. The phlebostatic axis is the anatomic projection of the right atrium to the patient’s thorax; it is at the mid-axillary line at the fourth intercostal space [44]. The level of the right atrium—that is very close to the level of the aortic root—is conventionally used as the reference level for most hemodynamic measurements [45]. If, however, the pressure at the circle of Willis should be monitored, the transducer must be elevated to the level of the base of the brain (external acoustic meatus). Before the measurement starts, the transducer has to be zeroed using the zeroing function of the monitor. This has to be done to ensure that the system displays a pressure of 0 mmHg when it is opened towards the atmosphere. For zeroing, the stopcock of the pressure transducer has to be opened towards the atmosphere while activating the zeroing function on the monitor. The zeroing is considered successful when the blood pressure tracing is a zero line at a pressure of 0 mmHg. After this procedure, the stopcock of the pressure transducer needs to be closed to the atmosphere. Whenever the vessel of interest of the patient is moving relative to the pressure transducer, a leveling maneuver has to be performed. Further zeroing maneuvers during the measurement are not required anymore.

**Fig. 2:**
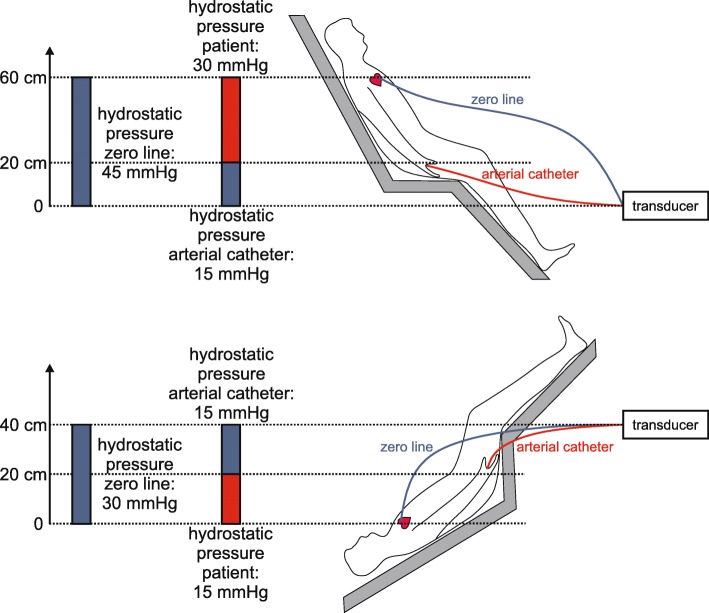
Arterial blood pressure measurement with a pressure transducer and a zero line. The free end of the zero line is attached to the body at the level of the vessel of interest. Before the start of the measurement, a zeroing maneuver is needed to account for the difference in height (i.e., hydrostatic pressure) between the free end of the zero line at the level of the vessel of interest and the transducer. Strictly speaking, this difference in height comprises two hydrostatic pressures (considering their theoretical mathematical signs, i.e., positive or negative): (1) the hydrostatic pressure caused by the height difference between the transducer and the arterial catheter and (2) the hydrostatic pressure caused by the height difference between the vessel of interest and the arterial catheter

Instead of adjusting the position of the transducer, one can also use a transducer and a zero line which is connected with the transducer. The zero line has to be completely filled with fluid; the free end of the zero line is attached to the body at the level of the vessel of interest. Before the start of the measurement, a zeroing maneuver is needed to account for the difference in height (i.e., hydrostatic pressure) between the free end of the zero line at the level of the vessel of interest and the transducer. Strictly speaking, this difference in height comprises two hydrostatic pressures (considering their theoretical mathematical signs, i.e., positive or negative): (1) the hydrostatic pressure caused by the height difference between the transducer and the arterial catheter and (2) the hydrostatic pressure caused by the height difference between the vessel of interest and the arterial catheter (Fig. 2). For zeroing, the stopcock of the pressure transducer has to be opened towards the zero line while activating the zeroing function on the monitor. During this procedure, the hydrostatic pressure which is caused by the fluid in the zero line and which always is the mathematical sum of the two hydrostatic pressures mentioned above is set as a pressure of 0 mmHg. After zeroing, the stopcock of the zero line needs to be closed. This zeroing maneuver has to be performed every time when the vessel of interest moves relative to the pressure transducer. Additional movements of the transducer (leveling) will lead to false BP measurements and therefore need to be avoided. Since the hydrostatic pressure of the fluid column in the zero line is used for zeroing, it is important that the zero line does not contain any air.

After leveling and zeroing of the system, a calibration of the pressure transducer can be performed, even though this maneuver is not frequently performed nowadays. Static calibration uses one or more predefined pressures to calibrate the pressures applied to the pressure transducer to the monitor output value. Dynamic calibration accounts for the frequency response (see step 5) of the measuring system. It can be performed using a sine-wave generator that mimics different input frequencies to the BP measuring system.

### Step 5: Checking the quality of the arterial blood pressure waveform—morphology and artifacts

Optimal quality of the BP waveform is fundamental to correctly measure BP and estimate derived hemodynamic variables [15, 46]. The performance characteristics of the measurement system are determined by the mass of fluid within the tubing system, the elasticity of the tubing system, and the friction between the fluid and the tubing system [6, 47]. The performance characteristics are quantitatively described by the natural frequency of the measurement system (the frequency of pressure pulse oscillations within the system) and the damping coefficient (describing the decay of the oscillating waveform) [6, 47]. The combination of the natural frequency and the damping coefficient determines the dynamic response of the arterial catheter/tubing/transducer system to the pulse pressure impulses coming from the cardiovascular system. Two main types of artifacts resulting from an inappropriate dynamic response can markedly alter the arterial waveform: underdamping (or resonance) and overdamping. Underdamping or overdamping are determined by the damping coefficient of the whole system, which can be seen as the entity of friction forces that oppose to oscillations.

The damping coefficient depends on several variables, especially the internal radius and the length of the catheter [15]. The longer the catheter, the higher is the damping coefficient. The larger the radius of the catheter, the lower is the damping coefficient and the higher the probability of recording an underdamped signal.

**Fig. 3:**
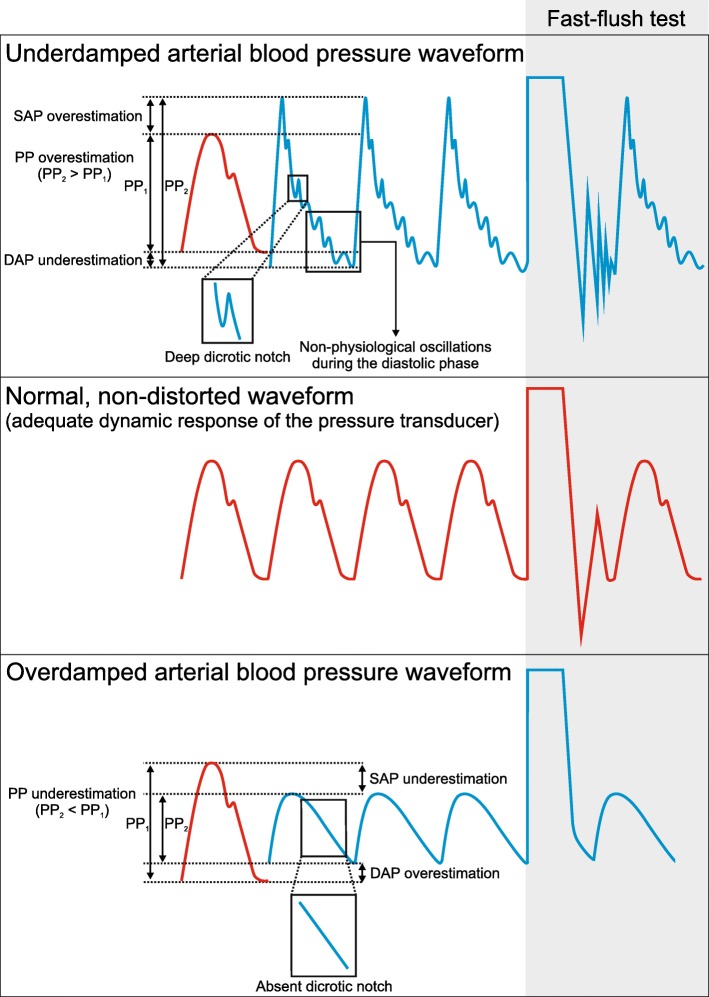
Under- and overdamping of the arterial blood pressure waveform and fast-flush test. The figure illustrates the characteristic changes of the arterial blood pressure waveform in case of under- and overdamping and the corresponding fast-flush test. The red arterial blood pressure waveform represents a “normal,” non-distorted waveform with normal fast-flush test, whereas the blue arterial blood pressure waveforms represent an underdamped (upper part of the figure) or overdamped (lower part of the figure) arterial blood pressure waveform. PP, pulse pressure; SAP, systolic arterial pressure; DAP, diastolic arterial pressure

In case of underdamping, the represented waveform differs from the true waveform as follows (Fig. 3):

- Systolic BP overestimation. A systolic pressure overshoot with a narrow peak can be observed (discrimination from the contribution of a reflected wave is the most complex aspect in this case).
- Diastolic BP underestimation.
- Pulse pressure overestimation.
- Deep dicrotic notch.
- Non-physiological oscillations during the diastolic phase.

The main reasons for an underdamped BP signal are excessively stiff tubing and a defective transducer [44].

In case of overdamping, the represented waveform will differ from the true waveform as follows (Fig. 3):

- Systolic BP underestimation.
- Diastolic BP overestimation.
- Pulse pressure underestimation.
- Slurred upstroke.
- Absent dicrotic notch.
- General loss of detail.

The main reasons for an overdamped BP signal are low infusion bag pressure, air bubbles in the circuit, blood clots, lose or open connections, and kinking or obstruction of the catheter [44].

**Fig. 4:**
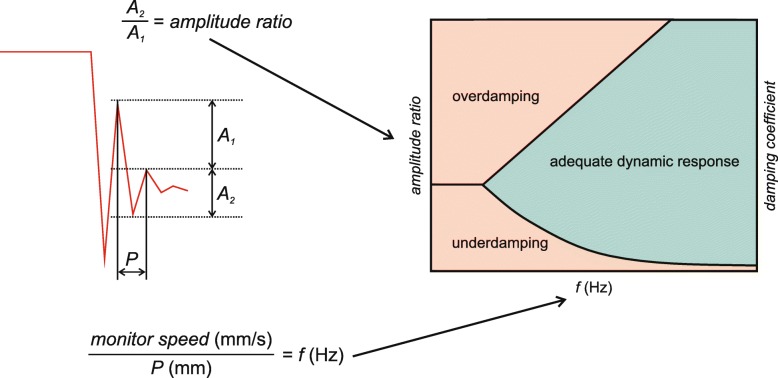
Quantification of the fast-flush test [47]. The figure illustrates how to interpret the fast-flush test. The natural frequency (*f*) of the system is calculated by dividing the monitor speed by the peak to peak distance (*P*) of the oscillating waves. Additionally, the amplitude ratio of two consecutive resonant waves has to be calculated by dividing the amplitude of the smaller wave (*A*_2_) by the amplitude of the higher one (*A*_1_). The amplitude ratio is then plotted against *f* in a specific graph that shows three areas: adequate dynamic response, overdamping, and underdamping

It is of crucial importance to visually check the BP waveform to ensure impeccable signal quality and identify artifacts. To test the damping properties of the measurement system, fast-flush tests should repeatedly be performed (Fig. 3) [47]. The fast-flush test (or square wave test) is performed by flushing the crystalloid fluid that fills the tubing/transducer system with 300 mmHg pressure via the flush system. This maneuver generates high amplitude oscillating waves that will fade exponentially after the flushing maneuver depending on the damping coefficient. The natural frequency of the system is calculated by dividing the monitor speed (e.g., 25 mm/s) by the wavelength (peak to peak distance in mm) of the oscillating waves (Fig. 4). Damping will change the amplitude of the oscillations, as it influences the energy in an oscillating system. Thus, the amplitude ratio of two consecutive resonant waves can be calculated by dividing the amplitude of the smaller wave (second) by the amplitude of the higher one (first). Once the amplitude ratio is calculated, it can be plotted against the natural frequency in a specific graph that shows three areas: adequate dynamic response, overdamping, and underdamping [15, 47]. In clinical practice, the oscillating waves induced by the fast-flush test usually are visually inspected.

To reduce the likelihood of underdamping, the operator should use short, stiff, non-compliant tubing; reduce movement of the catheter in the artery; and limit the number of stopcocks. A general recommendation is to not modify the transducer package (e.g., additional stopcock and/or extension lines) unless it is absolutely necessary for clinical purposes (e.g., positioning with arms along the body in the operating room) since the components of a transducer kit are carefully selected with the aim to find optimal physical properties to avoid artifacts due to over- or underdamping. If the transducer package has to be modified, it is important to only use extra lines and stopcocks that are made for BP measurement systems. If these precautions are insufficient, adjustable damping devices can be used to modify (increase) the damping coefficient of the system when underdamping affects the signal transmission.

In case of overdamping, since the main reasons are air bubbles or blood clots in the circuit, or kinking of the catheter, the only procedures potentially effective are modifying the wrist position in case of kinking, removing air or blood clots from the tubing, or changing the catheter and arterial site.

In case of under- or overdamping, the monitor scaling should be checked, as an inappropriate scaling can imitate an under- or overdamped BP signal.

Besides the abovementioned artifacts, patient movement during BP measurements or leaning against the patient’s arm used for BP monitoring may falsify BP reading. In addition, substantial variations in BP reading between different measurement systems may occur because different monitor devices use different algorithms for data processing, data averaging, and artifact filtering [48].

## Conclusions

Continuous invasive BP measurement using an arterial catheter is the clinical reference method for BP monitoring in high-risk surgical patients and critically ill patients. A key prerequisite for correct invasive BP monitoring is an in-depth understanding of the measurement principle and of BP waveform quality criteria. To correctly measure BP using an arterial catheter, we propose a systematic 5-step approach that helps to (1) choose the catheter insertion site, (2) choose the type of arterial catheter, (3) place the arterial catheter, (4) level and zero the transducer, and (5) check the quality of the BP waveform.

## Data Availability

Not applicable.

## Abbreviations

BP: Arterial blood pressure
G: Gauge
US: Ultrasound
