# Correction to: How to measure blood pressure using an arterial catheter: a systematic 5-step approach

**DOI:** 10.1186/s13054-020-03093-0

**Published:** 2020-06-23

**Authors:** Bernd Saugel, Karim Kouz, Agnes S. Meidert, Leonie Schulte-Uentrop, Stefano Romagnoli

**Affiliations:** 1 Department of Anesthesiology, Center of Anesthesiology and Intensive Care Medicine, University Medical Center Hamburg-Eppendorf, Martinistrasse 52, 20246 Hamburg, Germany; 2 Outcomes Research Consortium, Cleveland, OH USA; 3 Department of Anaesthesiology, University Hospital LMU Munich, Munich, Germany; 4 Department of Health Science, Section of Anesthesia and Critical Care, University of Florence, Florence, Italy; 5 Department of Anesthesia and Critical Care, Azienda Ospedaliero-Universitaria Careggi, Florence, Italy

**Correction to: Crit Care (2020) 24:172**

**https://doi.org/10.1186/s13054-020-02859-w**

In the publication of this article [1], there was an error in Fig. 4. The arrow indicating the amplitude A_1_ was too short and therefore did not cover the correct amplitude of the waveform.

The correct figure is:


